# Juices and By-Products of Red-Fleshed Sweet Oranges: Assessment of Bioactive and Nutritional Compounds

**DOI:** 10.3390/foods12020400

**Published:** 2023-01-14

**Authors:** Jaime Zacarías-Garcia, Guiselle Carlos, José-Vicente Gil, José Luís Navarro, Lorenzo Zacarías, María-Jesús Rodrigo

**Affiliations:** 1 Instituto de Agroquímica y Tecnología de Alimentos (IATA), Consejo Superior de Investigaciones, Científicas (CSIC), 46980 Valencia, Spain; 2 Food Technology Area, Faculty of Pharmacy, University of Valencia, 46100 Valencia, Spain

**Keywords:** red-fleshed orange juice, pasteurization, high-pressure homogenization, juice quality, bioactive compounds, antioxidant capacity

## Abstract

The content of nutrients and bioactive compounds, and antioxidant capacity were assessed in the juices from two red-fleshed oranges, Cara Cara and Kirkwood, and compared with that of a standard Navel orange. Two juice extraction procedures, hand-squeezing and industrial, and two treatments, pasteurization (85 °C/30 s) and high-pressure homogenization (HPH, 150 MPa/55 °C/1 min), were evaluated. For most of the nutrients and bioactive compounds, the hand and industrial juice squeezing rendered similar extraction efficiency. Individual composition of carotenoids in the juices were differentially affected by the extraction procedure and the treatments, but the red-fleshed orange juices contained between 3- to 6-times higher total carotenoids than the standard Navel juices, being phytoene and phytofluene the main carotenoids. The industrial and treated juices of both red-fleshed oranges contained 20–30% higher amounts of tocopherols but about 20% lower levels of vitamin C than Navel juices. Navel juices exhibited higher hydrophilic antioxidant capacity, while the red-fleshed orange juices showed an improved lipophilic antioxidant capacity. The main distinctive characteristic of the industrial juice by-product of the red-fleshed oranges was a higher content of carotenoids (×10) and singlet oxygen antioxidant capacity (×1.5–2) than the Navel by-product.

## 1. Introduction

Citrus is one of the main horticultural crops worldwide in terms of production and economic value. Spain produces over 50% of total citrus fruits in the EU and is the main exporter for fresh consumption. Other producer countries, such as Brazil, assign 90% of their production to the juice industry, while in Spain, about 80% of the total production is marketed as fresh, and nearly 20% is processed [1,2].

Citrus fruits and juices are widely consumed worldwide and are a natural source of micro- and macronutrients such as minerals, sugars, organic acids, vitamins C, E, and folates, and other bioactive phytochemicals such as carotenoids and phenolic compounds (including flavonoids and coumarins), among others [3,4,5]. Citrus fruit and juice consumption have been related to numerous beneficial effects on health, mainly attributed to the balanced content of nutrients and bioactive compounds [3,6,7,8,9].

Among the bioactive compounds present in citrus fruits and juices, carotenoids also determine their characteristic pigmentation [10,11]. Carotenoids are very efficient quenchers of singlet oxygen and scavengers of other ROS [12,13,14]. The carotenoid profile in the pulp and juice of standard blond sweet oranges can vary among varieties [15,16,17], but, in general, the β,β-xanthophylls content represents over 90% of total carotenoids, and linear carotenes are at low concentrations [18,19,20,21,22]. Within β,β-xanthophylls, violaxanthin is the predominant carotenoid, followed by lower amounts of lutein, antheraxanthin, zeaxanthin, and β-cryptoxanthin [19,23].

Accumulation of lycopene is an unusual characteristic that has been reported in the pulp of different species of *Citrus* and confers a bright pink/reddish coloration. To date, a limited number of red-fleshed orange varieties have been described: Shara [24], Cara Cara [25], and Hong Anliu [26]. Among them, Cara Cara (CC), a spontaneous mutation from the blond-fleshed orange Washington Navel, has been the best characterized [20,21,27,28,29]. Recently, a new red-fleshed orange, referred to as Kirkwood (K), which is a spontaneous mutation from Palmer Navel, was characterized for the first time [22,30]. The pulp of both red-fleshed varieties, CC and K, are characterized by a higher carotenoid concentration compared to standard orange-colored Navel varieties. The main feature of the carotenoid composition of CC and K is the outstanding amount of the colorless phytoene and phytofluene, low to moderate contents of lycopene and β-carotene, and reduced levels of violaxanthin compared to standard blond oranges [20,21,22,28,29,30]. Because of their exceptional coloration, carotenoids have been the most evaluated compounds in these red-fleshed oranges. Nonetheless, other studies have also assayed the concentration of other relevant compounds both in pulp and juice. Zacarías-García et al. [31] reported that the content of vitamin C, tocopherols, flavonoids, sugars, and organic acids in K pulp was essentially similar to that found in the standard variety Foios Navel. Nevertheless, contrasting results have been obtained in CC. Some studies found virtually the same content of vitamin C and flavonoids [32], whereas others reported minor amounts of vitamin C and total flavanones compared to standard Navel oranges [21,29].

In vitro and in vivo studies have investigated the potential health-related effects of the CC juice intake in comparison to the juice from standard varieties, reporting improved responses in antioxidant, inflammatory, and other relevant clinical parameters for the red-fleshed orange [33,34,35].

The citrus juice industry has developed several processing technologies to increase product shelf-life, ensure microbiological safety and preserve organoleptic attributes, nutrients, and bioactive phytochemicals of juices. However, juice processing and treatment technologies can affect both sensory and nutritional quality and the concentration of bioactive compounds to a variable degree. Traditionally, pasteurization has been used to assure microbiological safety and to inactivate enzymes such as pectin methylesterase (PME), which is responsible for the loss of turbidity, consistency, and gelation of orange juices [36,37]. In particular, pasteurization of red-fleshed orange juice from CC did not influence the total carotenoids and lycopene content [29,38], whereas β,β-xanthophylls levels were significantly affected [29]. On the other hand, high-pressure homogenization (HPH) is considered a promising alternative juice processing treatment for the commercialization of high-quality, healthy, and safe citrus juices [39]. HPH is considered a non-thermal technology where the fluid goes through a minute gap, which not only homogenizes the fluid but also increases the fluid’s temperature. This process reduces and homogenizes the particle size while inactivating microorganisms by minimally modifying the sensory and nutritional characteristics [39,40]. The effect of HPH treatment has been recently evaluated in citrus juices, and it seems a sustainable option to obtain juices with improved nutritional properties [39,41].

During the juice production process, only approximately half of the total weight of the orange fruit is transformed into juice, generating a large amount of waste [42]. The peculiar characteristics of citrus processing residues involve considerable limitations for their management due both to economic and environmental factors [43]. The waste of the orange juice industry is composed mainly of peel (flavedo and albedo), pulp, and seeds and is rich in fiber, essential oils, pectin, and antioxidants [44]. Citrus wastes have traditionally been used for animal feed and biorefinery industries. However, as innovative and more sustainable technologies are being developed, new uses for citrus by-products are emerging as added-value products for cosmetics, antifungals, biofuels, and fertilizers, among others [42,43,44].

This study aimed at evaluating the suitability for juice production of two red-fleshed orange varieties, CC and K, in comparison to a standard orange variety, Navel (N). To that end, we studied the effect of two juice extraction methods, freshly hand-squeezed and industrial, and two treatments, high-pressure homogenization and pasteurization, on the content of bioactive compounds (with a special interest in carotenoids), compounds related to organoleptic quality and the antioxidant capacity. Finally, we investigated the composition of the main bioactives and antioxidant capacity of the orange by-product of each variety generated during the industrial juice extraction process.

## 2. Materials and Methods

### 2.1. Plant Material

**Figure 1:**
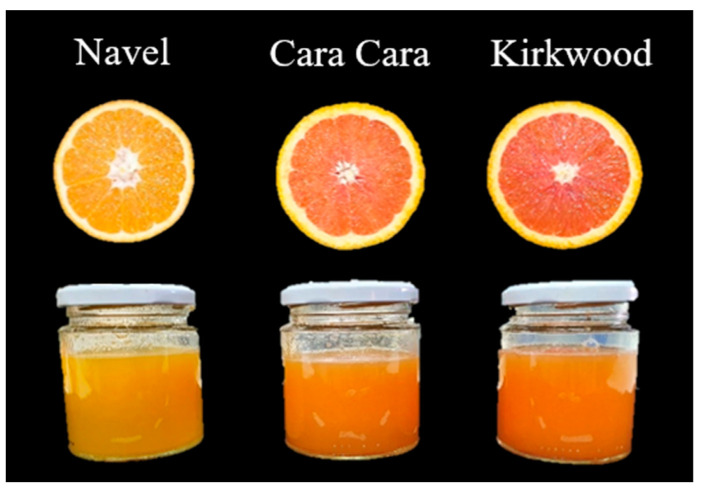
Appearance of pulp and freshly hand-squeezed juice of the sweet orange Navel and the red-fleshed Cara Cara and Kirkwood oranges.

Fruits of the three orange varieties (*Citrus sinensis* L. Osbeck) used in this study belong to the Navel group: two red-fleshed, Cara Cara (CC) and Kirkwood (K), and one standard Navel, Navel Foios (N). The standard N is a mid-season variety being one of the most cultivated varieties in Spain, and characterized by the intense orange color. CC and K are bud mutations characterized by the red coloration of the pulp due to an altered carotenoid composition [22,27,28]. Fruit of the red-fleshed varieties CC and K were harvested in commercial orchards from Ayamonte (Huelva, Andalucía, Spain), showing a maximum and minimum average temperature during the month of harvest (January) of 15.7 °C and 6.1 °C, respectively. On the other hand, the fruit of the blond-fleshed orange variety Navel was harvested in commercial orchards from Picassent (Valencia, Comunidad Valenciana, Spain) with a maximum and minimum average temperature during the month of harvest of 15.0 °C and 3.5 °C, respectively. (https://www.meteoblue.com/; https://www.aemet.es accessed on 9 January 2023). Fruits of all varieties were harvested at commercial maturity following the criteria described by Lado et al. [11]. Figure 1 illustrates the differences in pigmentation between the pulp and juice of the blond-fleshed N and the red-fleshed CC and K oranges.

### 2.2. Experimental Design

**Figure 2:**
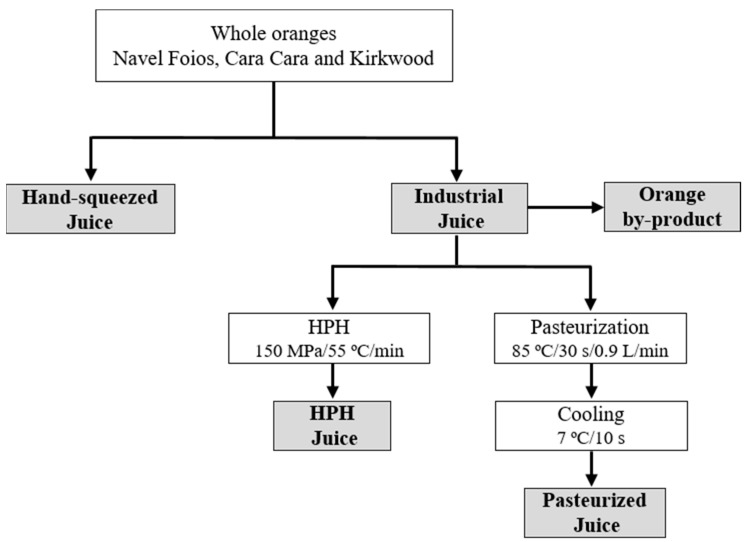
Flow diagram of the experimental design. HPH: high-pressure homogenization.

Immediately after harvesting, fruits were delivered to the laboratory and cold stored (2 °C) until processed in the next 3 days. For juice extraction, between 150 and 160 kg of fruits of each variety were used. The fresh hand-squeezed and the industrial juice and the treated (high-pressure homogenized and pasteurized) juices were obtained according to the experimental design shown in Figure 2.

Hand-squeezed juice (HS): This juice (approximately 10 L) was obtained by hand squeezing the fruits through a household electric hand reamer (Citromatic MPZ22, Braun, Barcelona, Spain) and subsequently filtered with a steel sieve of 2 mm to remove pulp.

Industrial juice: This juice was obtained through an industrial extractor (Exzel, Luzzysa; Valencia, Spain) and sieved in a paddle finisher (0.4 mm mesh diameter, model EPF 06, Luzzysa, Valencia, Spain). The industrial juice was split into two batches of approximately 25 L each for processing: one batch was pasteurized, and the other was high-pressure homogenized. 

Pasteurized juice: The pasteurization was performed at 85 °C for 30 s using a plate heat exchanger and cooled at 7 °C in the outlet section [39].

High-pressure homogenized juice (HPH): The industrial juice was processed at 150 MPa, reaching a temperature of 55 °C for 1 min. Homogenization was performed by a continuous system (NS3015H model, GEA Niro Soavi S.p.A., Parma, Italy) [39].

Orange by-product: The orange by-product consisted of peel (flavedo and albedo) and pulp remaining after industrial juice extraction. A sample of approximately 1 kg of each orange variety by-product was freeze-dried using a LYOBETA 6 PL (Telstar, Terrassa, Barcelona) for preservation and then stored at −80 °C until analyses. After freeze-drying, all orange by-products lost about 80% of their initial fresh weight.

### 2.3. Maturity Index and Juice Color

Total soluble solids (TSS, °Brix) and total titratable acidity (TA, mg of citric acid/100 mL of juice) were determined by using a digital refractometer PAL-BX/ACID1 (ATAGO, Japan). The maturity index (MI) was calculated as the TSS/TA ratio.

The juice color index expressed as the ratio *a/b* was analyzed using a CM-3500d spectrophotometer (Konica Minolta, INC., Japan). Color data were measured using the HunterLab scale, which defines the color in a three-dimensional space. The coordinate *L* indicates lightness, and a and b are green-red and blue-yellow coordinates, respectively. *L* is an approximate measurement of luminosity, taking values within the range of 0–100. The Hunter parameters a (assigns positive values for the reddish colors and negative values for the greenish ones) and b (assigns positive values to the yellowish colors and negative values to the bluish ones) were calculated and expressed as the ratio *a/b*, a classic relationship for color measurements in citrus fruits [45].

### 2.4. Carotenoids Determination

Carotenoids in the orange juice of the different varieties were extracted and analyzed essentially as described by Rodrigo et al. [20] with slight modifications. Briefly, 2 mL of orange juice was placed in screw-capped polypropylene tubes (15 mL), 3 mL of extraction solution composed of methanol:acetone:dichloromethane (25:25:50 *v*/*v*/*v*) was added, and the sample was sonicated for 5 min in an ultrasonic water bath at room temperature (XUBA3, Grant Instruments, Cambridge, England), centrifuged at 4500× *g* for 5 min at 4 °C (Labofuge 400R centrifuge, Thermo Scientific, Heraeus, Germany), and finally the organic phase was recovered. The aqueous phase was re-extracted with 1.5 mL of dichloromethane (HPLC grade, Sharlau, Barcelona, Spain) until the organic phase was colorless. The extracts were saponified in methanolic KOH (12%, *w*/*v*) for 90 min at room temperature and under a nitrogen atmosphere in darkness. After the saponification, 3 mL of 50 mM Tris-HCl pH 7.5 with 1 M NaCl and 3 mL of dichloromethane were added, stirred, and centrifuged at 4500 rpm for 5 min at 4 °C. The aqueous phase was discarded. This step was repeated (3 mL of Tris-NaCl buffer solution) until the discarded aqueous phase was neutral. The extracts were dried and kept at −20 °C until further analysis.

Carotenoids extraction and analysis was carried out from 0.25 g of freeze-ground dry orange by-product as essentially described by Zacarías-García et al. [22,31] for pulp and orange peel. 

The carotenoid composition was analyzed by high-performance liquid chromatography (HPLC) with a Waters liquid chromatography system equipped with a 600E pump coupled to a 2998 photodiode array detector (PAD) and Empower3 software (Waters, Barcelona, Spain). A C30 carotenoid column (250 × 4.6 mm, 5 μm) coupled to a C30 guard column (20 × 4.0 mm, 5 μm) (YMC, Tecknochroma, Barcelona, Spain) was used. Chromatographic conditions are described in Zacarías-García et al. [22,31]. The carotenoids were identified by absorbance spectra and retention time, peaks integrated at their individual maximal wavelength, and their contents were calculated using the appropriate calibration curves, as described elsewhere [22,31]. Two independent replicates of each sample were analyzed, and the results are expressed as mean ± standard deviation.

### 2.5. Vitamin C Determination

L-Ascorbic acid was extracted from 2 mL of orange juice and 0.2 g orange by-product and determined essentially as described in Alòs et al. [46] using a Waters Acquity Arc HPLC system (Waters, Barcelona, Spain) equipped with a DAD, Empower 3 software and an Ultrabase C18 column. Two independent replicates of each sample were analyzed, and the results are expressed as mean ± standard deviation.

### 2.6. Tocopherols Determination

Tocopherol extraction from orange juices and orange by-products was carried out essentially as described in Rey et al. [47] with slight modifications. Two mL of juice or 0.25 g of lyophilized orange by-product was extracted with 1.5 mL of methanol and 4 mL of dichloromethane, vortexed, and sonicated for 5 min. The samples were centrifuged at 3500 rpm for 10 min at 4 °C. The organic phase was recovered and extracted again with 2 mL dichloromethane.

Tocopherol content was determined by HPLC (Waters Acquity Arc system) coupled to a Waters 2475 FLR fluorescence detector and a YMC C30 column (150 × 4.6 mm, 3 μm) (Teknokroma, Barcelona, Spain) at room temperature. Chromatographic conditions used are described in Rey et al. [47]. The identification and quantification of tocopherols was achieved by comparison with the retention times of δ-, γ- and α-tocopherol standards (Sigma-Aldrich, Barcelona, Spain). Total tocopherol content was calculated as the sum of the different tocopherol isoforms. Two independent replicates of each sample were analyzed, and the results are expressed as mean ±standard deviation.

### 2.7. Analysis of Total Phenolic and Flavonoids

Total phenolic and flavonoid content was determined as described in Zacarías-García et al. [31] for orange pulps. Total phenolic was expressed as mg of gallic acid equivalents (GAE) per 100 mL of juice or 100 g of dry weight. Total flavonoids were analyzed in the orange by-products and expressed as mg of hesperidin equivalents (HesE) per 100 g of dry weight. Determinations were performed per triplicate in each extract. 

### 2.8. Flavonoid Analysis by HPLC-DAD

The extraction of flavonoids from 2 mL of orange juice was performed according to the procedure described in Zacarías-García et al. [31]. Flavonoid composition was determined by HPLC-DAD (Waters Acquity Arc system). Separation of flavonoids was carried out with an XBridgeTM BEH C18 column (4.6 × 150 mm, 2.5 µm Column XP). Chromatographic conditions are described in Zacarías-García et al. [31]. Identification and quantification of the different flavonoids was achieved by comparison with the retention times and peak areas of authentic standards of hesperidin, narirutin, naringin, eriocitrin, dydimin, and rutin (Sigma-Aldrich, Barcelona, Spain). Two independent replicates of each sample were analyzed, and the results are expressed as mean ± standard deviation. 

### 2.9. Sugars and Organic Acids Determination

Sugars and organic acids were extracted and determined essentially as described in Zacarías-García et al. [31]. Sugars and organic acids content was measured by HPLC (Thermo Fisher Scientific, Waltham, MA, USA) equipped with a refraction index detector. For glucose, fructose, citric acid, malic acid, quinic acid, and succinic acid, separation and determination were employed in a HyperREZTM XP Carbohydrate H+ 8 μm column (Thermo Fisher Scientific). For sucrose determination, a Pb column was used (Hi-Plex Pb, 300 × 7.7 mm, Agilent Technologies). Chromatographic conditions used are described in Pérez-Través et al. [48]. The identification and quantification of sugars and organic acids were carried out by comparison with the retention times of authentic standards (Sigma-Aldrich, Barcelona, Spain). Two independent replicates of each sample were analyzed, and the results are expressed as mean ± standard deviation.

### 2.10. Determination of Antioxidant Capacity

Three different methods were performed for the antioxidant capacity of the orange juices and by-products. The hydrophilic antioxidant capacity (HAC) of juices (2 mL) and orange by-product (0.25 g DW) was determined using DPPH free radical assay (2,2-diphenyl-1-picrylhydrazyl) [31], FRAP (ferric reducing antioxidant power) [31] and radical ABTS (2,2′-azino-di-(3-ethylbenzthiazoline sulfonate)) [31]. The lipophilic antioxidant capacity (LAC) of orange juices and by-products was also determined by ABTS [31]. Each sample was analyzed in two independent assays, and samples were run in triplicate in each plate.

### 2.11. Singlet Oxygen Absorption Capacity (SOAC)

The singlet oxygen absorption capacity (SOAC) of orange by-products was determined according to the procedure previously described [31,49,50]. The relative SOAC value for each sample was calculated with the following formula (1):

(t1/2 sample − t1/2 blank)/(t1/2 α-toc − t1/2 blank) × ([α-toc, g/L]/[sample, g/L])(1)

Sample refers to an extract of 0.3 g dried pulp tissue in 6 mL of ethanol:chloroform:water (50:50:1), while blank is only a mixture of ethanol:chloroform:water (50:50:1, *v*:*v*:*v*).

### 2.12. Retinol Equivalents

The capacity pro-vitamin A expressed as retinol equivalents (RE) was calculated following the equation of Sanchez-Moreno et al. [51]

RE = µg β-carotene/6 + µg α-carotene/12 + µg β-cryptoxanthin/12(2)

### 2.13. Statistical Analysis

Results are the mean of two independent replicates ± standard deviation (SD). One-way ANOVA was carried out, and Tukey’s test (significance level *p* ≤ 0.05) was used for mean comparisons among varieties and treatments. Principal component analysis (PCA) was set up using the compound’s concentration and antioxidant values. Analysis was made using the XLSTAT Software version 2019.3.2 (Addinsoft, Paris, France). The correlation matrix between bioactive compounds concentration and antioxidant capacity was carried in RStudio (version 1.3.1093, RStudio Team, PBC, Boston, MA, USA) using the function “cormat” and visualized using the function “corrplot” of the package “ggplot2” [47].

## 3. Results and Discussion

This study aimed at evaluating the potential of red-fleshed orange varieties for juice production and how juice processing affects main bioactive and quality compounds. To that end, hand-squeezed (HS), industrial pasteurized (85 °C/30 s), and non-thermal high-pressure homogenization (HPH) (150 MPa/55 °C/1 min) juices were prepared from fruits of the red-fleshed orange (*Citrus sinensis*) varieties Cara Cara (CC) and Kirkwood (K), and from fruits of the standard orange variety Navel (N). The juice yield for the three varieties ranged from 42–45% (*w*/*v*). Industrial orange juice extraction generates great amounts of fruit wastes of high potential interest for food/feed, pharmaceutical, and cosmetic sectors or for bio-transformation processes. Thus, to investigate the potential value of red-fleshed orange by-products in comparison to that of the traditional variety N, a characterization of the bioactive compounds was also carried out.

### 3.1. Maturity Index and Orange Juice Color

The maturity index is a reference quality parameter in citrus fruits and is determined by the ratio between the content of soluble solids (°Brix) and acidity [11]. Juices of CC presented lower °Brix levels than N and K fruits (Table 1). However, no significant differences in acidity and in maturity index were observed between the three varieties (Table 1). Therefore, our results showed that the red-fleshed oranges CC and K reached a similar maturity index to the standard N at a mature stage. Other works reported that fruits of Navel orange presented slightly higher levels of °Brix and maturity index compared to CC [20,21], but these differences are more likely attributed to variability in the ripening stage at harvest and/or the environmental and agronomic conditions [11].

The color represents one of the most important features of citrus fruit quality, and it is a crucial issue for consumer acceptance. Moreover, the utility of the Hunter color parameters (*L* and *a/b*) to classify different orange juices according to the industrial processing requirements has been previously reported [52]. The CC and K fruits stand out by the reddish coloration of the pulp, while fruits of N present the typical orange tone of traditional oranges [20,21,22,28]. The visual inspection of CC and K juices showed a more intense orange-reddish coloration than in N juices, regardless of the extraction procedure (hand-squeezed or industrial) and the treatment applied (HPH or pasteurization). This color perception corresponded to a significantly higher *a/b* ratio and lower *L* values of CC and K juices compared to N (Table 2). It was noted that the HS juice of K exhibited a slightly higher color index than CC. The method of juice extraction exerts a notable effect on the *a/b* ratio. Thus, the HS juice of all varieties displayed a higher *a/b* ratio and lower *L* values than the corresponding industrial juice (Table 2). However, no differences were detected between the industrial and the processed juices or between the treatments for any variety (Table 2). The carotenoids responsible for the color of common orange juice are the xanthophylls, mainly the isomers of violaxanthin, antheraxanthin, zeaxanthin, and lutein [18], while the juice color of the red-fleshed oranges CC and K is strongly influenced by the concentration of lycopene [20,21,22,29]. Strikingly, the industrial juice of the three varieties evaluated displayed a lower color index (*a/b*) but higher lightness (*L*) compared to their respective HS juices (Table 2).

Nevertheless, the industrial juices contained either similar or higher total carotenoid content than the HS juices, as reported in the next section. These results might be explained by the modification of the pulp structure, and the particle size since industrial extraction produces greater mechanical stress and contributes to a higher dispersion of the particles resulting in lower pigmentation [53]. Stinco et al. [52]) correlated the smaller particle size of the industrial orange juices with brighter (higher *L*) and more yellowish (lower *a/b*) color than HS juices. Therefore, the lighter color detected for the industrial juices compared to those obtained by hand extraction agrees with previous studies [52]. According to these results, it could be concluded that the main differences in color between the HS and the industrial juices are more likely related to the extraction procedure than to the treatments, as it was previously proposed [52].

### 3.2. Carotenoids

The concentration of individual and total carotenoids of HS, industrial, HPH, and pasteurized juices of N, CC, and K are shown in Table 3. The total content of carotenoids in both red-fleshed orange juices ranged from nearly 25 to 35 µg/mL, while in N was approximately between 6 and 9 µg/mL; thus, the HS (3.4- and 4.0-fold), industrial (4.0- and 3.4-fold), HPH (5.4- and 5.8-fold) and pasteurized (5.7- and 5.2-fold) juices of CC and K contained more carotenoids than the corresponding juices of N, respectively. These differences in the total carotenoids among the juices of the red-fleshed and the ordinary orange varieties are in accordance with previous studies [20,21,29,34]. The carotenoid profile of N juices is primarily composed of β,β-xanthophylls, which provide the typical yellow-orange coloration. In the juice of this variety, the major carotenoid was violaxanthin (4.25 µg/mL), which represents 43–54% of the total carotenoids content, and other relevant β,β-xanthophylls were antheraxanthin (8–13%) (1.00–0.52 µg/mL), and β-cryptoxanthin (8–11%) (0.91–0.65 µg/mL) while low concentrations (less than 0.5 µg/mL) of zeaxanthin and the colorless carotenes phytoene and phytofluene were detected. Luteoxanthin and mutatoxanthin were also detected since the natural acidity of the juice promotes the rearrangement of 5,6-epoxy carotenoids, such as violaxanthin and antheraxanthin, to their respective 5,8-epoxy carotenoids [54]. The carotenoid profile of N juices is in consonance with that described for other Navel varieties [18,19,20,21,29]. On the other hand, the carotenoid composition of both red-fleshed orange juices was completely different to that of N and resembled that described for CC juice in a previous analysis [20,21,29] and the pulp of K [22,31]. The juice of CC and K oranges was characterized by high levels of phytoene (14.99–23.12 μg/mL and 18.99–22.14 μg/mL, respectively) and phytofluene (3.48–5.59 μg/mL and 3.99–4.89 μg/mL, respectively), accounting for 70–80% of total carotenoids (Table 3). The concentration of lycopene in the different juices varied between 1.30–2.30 μg/mL and 1.49–2.82 μg/mL in CC and K, respectively. It is also relevant to mention the reduction (40–50%) in the concentration of violaxanthin found in HS and industrial juices of CC and K compared to N. Interestingly, these differences were less relevant in HPH and pasteurized juices (Table 3). Besides, β-carotene was detected at low levels in CC (0.23–0.47 μg/mL) and K juices (0.29–0.45 μg/mL), while it was not detected in N. On the other hand, provitamin A capacity expressed as retinol equivalents (RE) was determined by the precursors detected in orange juices: β-carotene, which shows the highest vitamin A activity, and β-cryptoxanthin which is assumed to have half provitamin A activity than β-carotene [52]. Traditionally, β-cryptoxanthin has been considered the main source of provitamin A in citrus juices since levels of β-carotene are very low or not detected. Nonetheless, the RE determined in CC and K juices was nearly twice than that of N due to the higher concentration of β-carotene (Table 3). Therefore, CC and K represent a better source of provitamin A than the traditional Navel oranges.

Even though the different juices of each variety presented similar qualitative compositions of carotenoids, the method of juice extraction and treatment affected the content of carotenoids unevenly, depending on the orange genotype (Table 3). The industrial juice of N contained 18% more total carotenoids than the HS juice because of the larger content of phytoene, β-cryptoxanthin, luteoxanthin, and mutatoxanthin. Phytofluene was not detected in the HS juice of N. However, industrial extraction favored its extractability, although at very low levels (Table 3). Similarly, the industrial juice of CC presented 32% more total carotenoids compared to the HS due to the amounts of phytoene (34% more) and nearby the double concentration of lycopene. Contrastingly, the content and composition of carotenoids in K juices hardly varied between the two extraction methods. Industrial extraction has been shown to increase the content of carotenoids in orange juice since a more powerful squeezing of the fruits enhances the carotenoids release from the membranes of the pulp vesicles to the juice [55,56,57]. On the contrary, Stinco et al. [52] described that industrial processing exerts a negative impact on the content of carotenoids in the juice. These discrepancies may be attributed to the use of different orange varieties and the impact of the processing conditions, which can have a significant influence on the carotenoid content.

Regarding the effects of the industrial juice treatments, HPH and pasteurization produced a notable reduction of the total carotenoid content in N juices, especially in β,β-xanthophylls, as β-cryptoxanthin, antheraxanthin, violaxanthin, and luteoxanthin, with around a 30% reduction respect to the industrial juice (Table 3). It has been classically described that thermal treatments negatively affect the concentration of carotenoids in orange juices since high temperatures during thermal processing might cause the instability of the polyene chain of the carotenoids, resulting in their degradation by isomerization, oxidation, and cleavage [29,41,58,59]. Our results agree with those of Etzbach et al. [60], who observed a significant reduction of violaxanthin in orange juice treated by conventional pasteurization (90 °C/30 s). Nevertheless, in that study, no changes in non-epoxy carotenoids such as lutein, zeaxanthin, and β-cryptoxanthin nor in monoepoxides as antheraxanthin were detected, suggesting that diepoxy carotenoids such as violaxanthin and luteoxanthin were more susceptible toward thermal degradation. On the other hand, in HPH and pasteurized juices of CC, the concentration of violaxanthin was also significantly reduced, while the levels of β-cryptoxanthin and zeaxanthin increased. Furthermore, the total carotenoid content remained relatively stable since the predominant carotenoids correspond to linear carotenes, which were not affected by the treatments. Variable observations concerning the effects of high-pressure treatments, high-pressure processing (HPP) and HPH, and pasteurization on the carotenoid content in orange juice are reported in the literature. In this sense, discrepancies might be attributed to the different processing conditions, such as juice extraction procedure, temperature, and equipment [37]. De Ancos et al. [21] showed that the HPP treatment at 200 and 400 MPa had a negative effect on the content of vioxalanthin and lycopene in Navel and CC juices, respectively. Sentandreu et al. [39] observed a significant decrease in total carotenoids in HPH-treated Navel juices.However, Lu et al. [38] did not observe changes in the content of carotenoids in CC juice after thermal treatment. The current study is the first evaluating the effect of processing on the juice from the red-fleshed orange K. We found that total carotenoids in HPH and pasteurized juices of K increased by 15% and 8%, respectively, compared to the industrial juice (Table 3). These effects were associated with a higher concentration of phytoene and phytofluene (9–19%) and of lycopene content (45%) (Table 3). It is worth mentioning that these carotenes are greatly hydrophobic, and HPH and pasteurization treatments may severely disrupt suborganellar cell structures where carotenes are accumulated [61,62] and then enhance their release to the juice. Nevertheless, other studies in CC and the yellow-pigmented orange variety, Pinalate, reported that HPH and pasteurization decreased the content of colorless carotenes and lycopene [29,41,62]. Therefore, it is likely that the effect of juice processing treatment on carotenoids may be related to the cellular deposition structures where the carotenoids accumulate in the chromoplasts, which may vary depending on their specific composition and contents [61,62].

In general, industrial extraction increased or maintained the concentration of carotenoids in the juice compared to hand-squeezing. Therefore, the industrial procedure may be considered a good option to optimize carotenoid content in the juice of the red-fleshed varieties without no detrimental effects. Additionally, HPH and pasteurization treatments have similar effects on carotenoid levels in industrial juices of these varieties, suggesting that temperature, rather than high pressures, is the main factor influencing carotenoid concentration in the juice [39,52,63]. The thermal stability of carotenoids is associated with their chemical structure, solubility, and hydrophobicity [64]. Hence, the variability of the results obtained by the HPH and pasteurization treatments might be explained by the differences in the matrix composition and in the structures of carotenoid storage between the orange varieties [21,22,29,61].

### 3.3. Vitamin C

**Figure 3:**
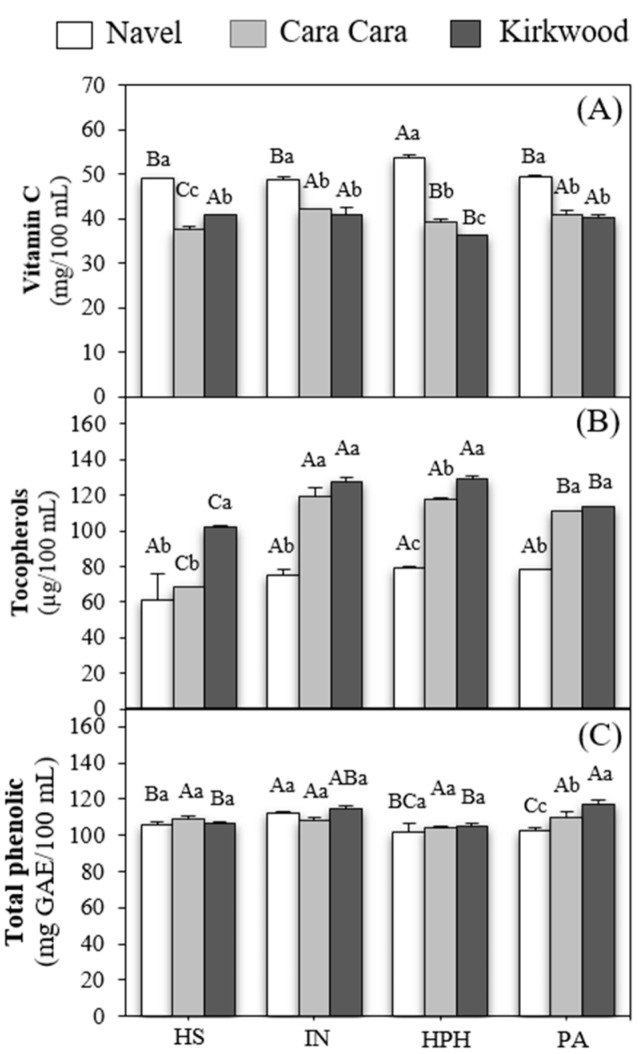
Content of vitamin C (**A**), tocopherols (**B**), and total phenolics (**C**) in hand-squeezed (HS), industrial (IN), HPH, and pasteurized (PA) juices of N, CC, and K orange. Uppercase letters indicate significant differences between different type of juices for the same variety, and lowercase letters indicate significant differences between varieties for the same type of juice by one-way ANOVA (*p* < 0.05).

All the juices of N orange presented 10–20% higher vitamin C concentration than those of CC and K (Figure 3A). This result is in good agreement with previous studies [21,38,65] suggesting that the genotype modulates the concentration of vitamin C [46]. However, recent studies accounted that the pulp of mature fruits of N and K contains similar values of vitamin C [31], which may indicate that vitamin C extractability from the pulp of red-fleshed oranges during juice processing is lower compared to standard oranges. Moreover, other factors such as the ripening stage of the fruit and the growing conditions may also be determinants of the vitamin C content and may result in differences between standard and red pulp oranges. In general, the extraction method and treatments applied to the industrial juices did not substantially affect the content of vitamin C of the three varieties, although some minor changes were detected. The industrial extraction in CC juice slightly incremented (6%) the vitamin C levels compared with the HS juice, while no differences between the extraction methods were detected in N and K. Gil-Izquierdo et al. [55] reported that Navel orange juice produced by commercial squeezing contained 25% more vitamin C than domestic squeezing. The HPH treatment increased the content of vitamin C in N juice, while it decreased in the CC and K juices compared to the industrial fresh juice. Similarly, other authors reported that high-pressure treatments exert variable effects on the concentration of vitamin C in orange juices [66,67], while others claimed that these types of treatments did not modify the concentration of this vitamin [21,59]. It has been suggested that only high-pressure treatments that entail a substantial temperature increment negatively affect vitamin C content due to its degradation or thermal oxidation [66]. However, pasteurization, which involves higher temperatures than HPH, did not reduce the concentration of vitamin C [55,68,69]. Taken together all these results, it is reasonable to suggest that discrepancies in the stability of vitamin C observed in the different studies depend on the combination of multiple factors, such as juice extraction and the conditions of the treatments (temperature, pressure, time, etc.) [21].

### 3.4. Tocopherols

The study of accumulation and biosynthesis of tocopherols in sweet oranges has received little attention to date [47,70,71]. To our knowledge, only a previous study has evaluated the concentration of tocopherols in red-fleshed oranges [31], and this is the first work analyzing tocopherols in juices of red pulp varieties. α-Tocopherol was the only isoform identified in all juices, with levels ranging from 61–129 μg/100 mL (Figure 3B). These values are very similar to those described for orange juices of standard varieties [70,71]. The analysis of tocopherols content showed that all juices of the red-fleshed varieties, except the hand-squeezed juice of CC, contained concentrations around 20–30% higher than those of N (Figure 3B). However, previous data showed similar concentrations of tocopherols in the pulp of mature fruits of K and N [31]. The industrial extraction compared to HS juices increased by 43% and 20% of the content of tocopherols in CC and K juices, respectively, but not in N (Figure 3B). These data suggest that juice extraction, and specifically industrial extraction, might enhance the extractability of tocopherols from the pulp vesicles to the juice matrix in the red-fleshed oranges. Pasteurization slightly reduced the levels of tocopherols in CC and K compared to the industrial and HPH juices (Figure 3B). Minor information is available on the stability of tocopherols during pasteurization and high-pressure processing in orange juices. Even though tocopherols are considered heat-sensitive compounds, contrasting results about their stability in different food matrices might be found. Cilla et al. [72] showed that HPP/400 MPa did not affect the concentration of α-tocopherol in milk-fruit beverages, but strikingly, heat treatment (90 °C/30 s) significantly increased its content. Instead, other works detected a significant reduction of α-tocopherol in human milk after pasteurization [73]. Overall, processing conditions, as well as food matrix characteristics, appear to be crucial factors for tocopherols’ stability after thermal treatments.

### 3.5. Phenolics and Flavonoids

The content of total phenolics experienced minor differences between varieties and treatments (Figure 3C). Only pasteurized juices displayed differences in total phenolics content between varieties in the following order K > CC > N. In agreement with our results, Brasili et al. [29] and De Ancos et al. [21] found similar total phenolic content in the juice of the Navel compared to CC by Folin-Ciocalteu assay. Similarly, in a previous study, we did not detect differences in either level of phenolics or flavonoids in the pulp of freshly harvested K and N fruits [31]. Additionally, our results agree with those of Bai et al. [56], that reported no differences in total phenolics between HS and industrial juices. Thus, a disparity in the effect of pasteurization on the levels of phenolics in orange juices is apparent. For example, Brasili et al. [29] described no effect of heat treatment on Navel and CC juices, while Bai et al. [56] supported that pasteurization increased total phenolic in orange juices.

The profile of flavonoids of N, CC, and K juices was analyzed by HPLC-DAD (Table 4). In general, all juices showed similar flavonoid composition. The flavanone glycosides hesperidin (56–70% of the total) and narirutin (17–29% of the total) were the major flavonoids found in all juices, accounting both as much as 85–89% of total flavonoids (Table 4) consistent with previous data for other orange juices [74]. Other less abundant flavonoids detected in all juices were rutin, eriocitrin, naringin, and dydimin. The concentration of total and individual flavonoids was similar between the juices of standard and the red-fleshed orange (Table 4). Previous studies indicated that Navel juices contained slightly higher flavonoid content than CC [29,32]. However, other studies did not find significant differences between the pulp [31] and juice [21] of blond and red-fleshed oranges. These discrepancies might be attributed to the influence of the maturity stage or the environmental and growing conditions in the levels of these phytochemicals. The comparison of the two juice extraction methods revealed that the hand-squeezing provided more flavonoids than the industrial extraction in the three varieties, although differences were only statistically significant in N and CC juices (Table 4).

This difference was mainly associated with the higher concentration of hesperidin in the HS juices. Regarding the processing juice treatments, neither the pasteurization nor the HPH treatment led to remarkable changes in the flavonoid content, in agreement with other citrus juice after HPH [39,59,63] and pasteurization [29,38,55,56,69].

### 3.6. Antioxidant Capacity

**Figure 4:**
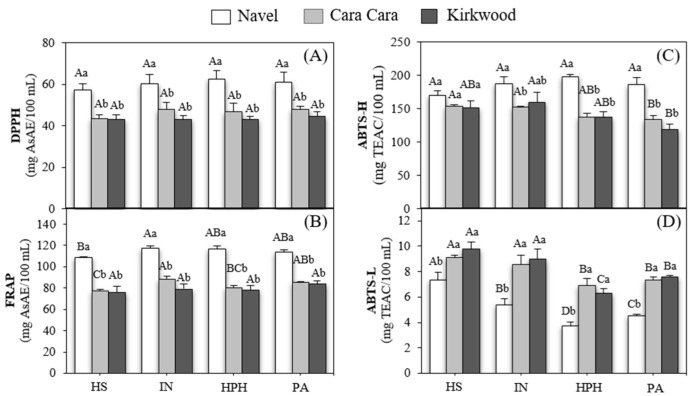
Hydrophilic antioxidant capacity assayed by DPPH (**A**), FRAP (**B**), and ABTS (ABTS-H) (**C**) and lipophilic antioxidant capacity assayed by ABTS (ABTS-L) (**D**) in hand-squeezed (HS), industrial (IN), HPH and pasteurized (PA) juices of N, CC and K orange. Uppercase letters indicate significant differences between different type of juices for the same variety, and lowercase letters indicate significant differences between varieties for the same type of juice one-way ANOVA (*p* < 0.05).

**Figure 5:**
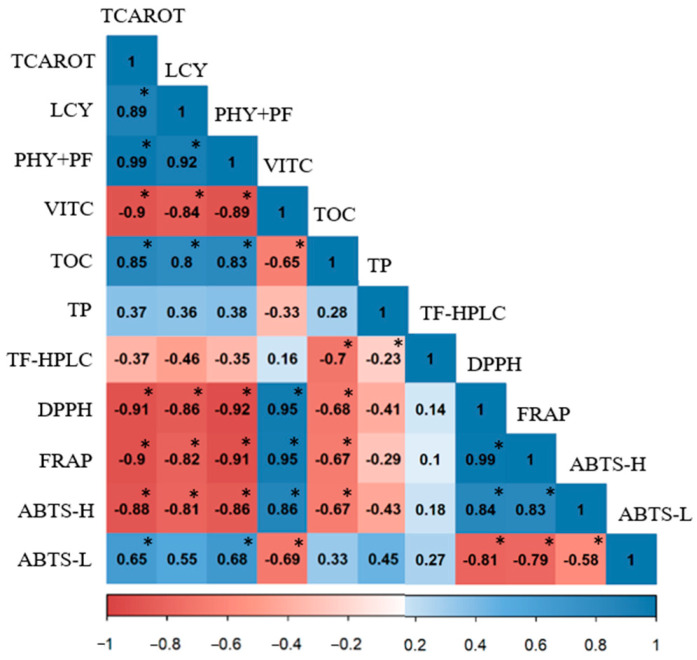
Matrix correlation between total carotenoids (TCAROT), vitamin C (VITC), tocopherols (TOC), total phenolics (TP), total flavonoids (TF), total flavonoids by HPLC (TF-HPLC) and antioxidant capacity (DPPH, FRAP, ABTS-H, and ABTS-L) values obtained in the different juices of N, CC and K. Positive and negative correlations are shown in different shades of blue and red, respectively. Significant Pearson’s correlation coefficient (*p* < 0.05) is indicated with an asterisk.

Hydrophilic antioxidant capacity (HAC) of N, CC, and K juices was evaluated by DPPH (Figure 4A), FRAP (Figure 4B), and ABTS (Figure 4C). The HAC showed similar results by the three methods assayed, and the N juices exhibited higher HAC than CC and K. Besides, no differences between both red-fleshed varieties were found for any type of juice. Positive correlations between DPPH and FRAP (r^2^ = 0.99), ABTS-H and DPPH (r^2^ = 0.84), and ABTS-H and FRAP (r^2^ = 0.83) were obtained (Figure 5). These findings were similar to previous results reporting an enhanced HAC in Navel juices in comparison with CC [21,29]. Nonetheless, Zacarías-García et al. [31] indicated that the HAC of the pulp of N and K fruit was similar. The analysis of correlations carried out between the bioactive compounds and antioxidant capacity display high positive correlation values between vitamin C content and DPPH (r^2^ = 0.95), FRAP (r^2^ = 0.95), and ABTS-H (r^2^ = 0.86) (Figure 5). On the contrary, total phenolic compounds (TP) and flavonoids (TF) that were also at high concentrations in the water-soluble fraction of orange juices did not show significant correlations with any of the antioxidant assays. These results agree with data provided in other studies, which indicate that vitamin C is the major contributor to the hydrophilic antioxidant capacity of orange juices [29,55,75,76].

The lipophilic antioxidant capacity (LAC) was determined by ABTS (referred to as ABTS-L) (Figure 4D) since this method can determine the antioxidant capacity in the lipophilic fraction of the juice extracted with organic solvents [76]. The ABTS-L indicated that CC and K juices presented a remarkably major capacity than N juices (15–40%) (Figure 4D). The ABTS-L showed a good significant correlation with the concentration of total carotenoids (r^2^ = 0.65) and the sum of phytoene and phytofluene (r^2^ = 0.68), but lower than with the content of lycopene (r^2^ = 0.55) (Figure 5). Even though lycopene has been described as the carotenoid with the highest antioxidant activity against ROS [12,77], the major lipophilic capacity of CC and K juices compared to N might be associated with the high levels of phytoene and phytofluene, which antioxidant properties have been also reported [13].

Therefore, the contribution of carotenoids to the LAC of juices depends not only on the total content but also on the individual composition of carotenoids [75,76]. However, other studies suggested that the contribution of carotenoids to the in vitro antioxidant capacity in orange juices is not relevant [21,75,78]. These discrepancies could be explained by the fact that the assays used to quantify this activity did not study independently the antioxidant capacity of the lipo- and hydrophilic fractions, and then, most of the results did not reflect the contribution of fat-soluble compounds [76,79,80]. On the other hand, despite the low correlation of tocopherol content with the lipophilic capacity (r^2^ = 0.33), it cannot be excluded the certain contribution of tocopherols to the antioxidant capacity of the juices.

The effect of the extraction method and treatment (HPH and pasteurization) on the antioxidant capacity of juices required an independent analysis. The hand and industrial squeezing demonstrated almost the same effectiveness in terms of antioxidant capacity [55]. The HAC of the juices of the three varieties was hardly affected by the treatments. Nevertheless, the LAC in the HPH and pasteurized juices of all varieties decreased in comparison to the corresponding industrial juice. Intriguingly, this reduction was not associated with a minor content of lipophilic compounds such as carotenoids and tocopherols in HPH and pasteurized juices (Table 3; Figure 3). Other authors also reported that thermal treatments might negatively affect LAC [81]. Thus, our data suggest that the reduction in LAC in treated juices could be associated with a decrease in the activity of other lipophilic compounds not analyzed in this work. De Ancos et al. [21] observed that HPP/400 MPa treatment reduced the antioxidant capacity in hydrophilic extracts of Navel and CC juices. In contrast, other authors did not detect differences in the antioxidant potential of orange juices after processing [55,59] or even observed an increase in the antioxidant capacity in CC juice [29]. The variability of results regarding the effects of thermal and non-thermal treatments on antioxidant capacity might be closely linked to the differences in the composition of the juices, processing conditions, type of extract (hydro- or lipophilic), and the antioxidant assay employed [37,82]. Together, the results obtained by the in vitro antioxidant assays, especially those of lipophilic, revealed promising perspectives of the antioxidant capacity of the red-fleshed varieties. Due to the limitations of the assays to determine the antioxidant capacity of the components of a complex food matrix, it would be of special interest to evaluate in future studies the biological effects of the juice from red-fleshed oranges by using in vivo systems and to corroborate the contribution of the high content of carotenoids detected in these oranges to the antioxidant activity.

### 3.7. Sugars and Organic Acids

**Figure 6:**
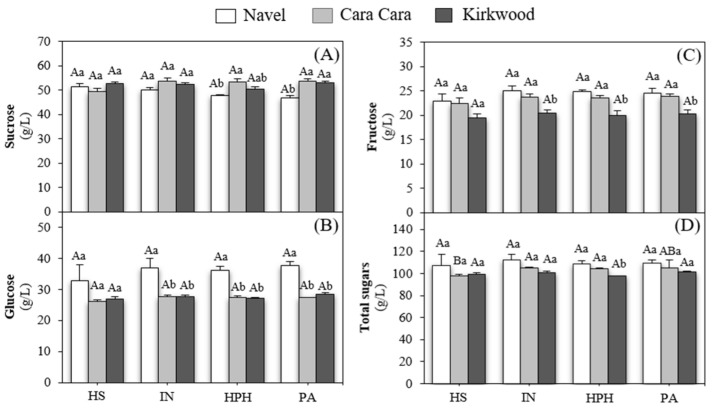
Content of sucrose (**A**), glucose (**B**), fructose (**C**), and total sugars (**D**) in hand-squeezed (HS), industrial (IN), HPH, and pasteurized (PA) juices of N, CC, and K orange. Uppercase letters indicate significant differences among different type of juices for the same variety, and lowercase letters indicate significant differences among varieties for the same type of juice one-way ANOVA (*p* < 0.05).

Sugars and organic acids are key compounds in the sensory properties of fruit juices [83]. Sucrose, glucose, and fructose were the main sugars in all juices (Figure 6). Sucrose was the most abundant (45.70–52.92 g/L), followed by glucose (25.48–37.04 g/L) and fructose (22.06–25.12 g/L), in a ratio near 2:1:1 (sucrose:glucose:fructose), which is a recognized parameter to identify genuine fresh orange juice from adulteration [58]. The concentrations of sugars detected in all juices were in the range described in the literature for orange juices [3,65]. The HS juice of the three varieties contained a very similar concentration of sugars (Figure 6). The HPH and pasteurized juices of CC and K contained slightly higher sucrose than N (Figure 6A). The content of glucose was 20–30% higher in N juices compared to the red-fleshed varieties (Figure 6B), and the levels of fructose were higher in N and CC than in K juices (Figure 6C). Overall, the total content of sugars was approximately equivalent in the juices of the three varieties (Figure 6D). Some authors reported lower concentrations of sugars in the juice of CC than those presented in this work [29,84]. Other studies obtained similar sugar concentrations in fruits of CC and K orange in comparison with N [29,31,65]. Moreover, HPH and pasteurization had no significant effect on the stability and composition of sugars in the juices of the three varieties, as reported previously [29,85].

Overall, the juices from the red-fleshed varieties presented slightly higher sucrose and lower glucose concentrations. Furthermore, the juices of N and CC contained larger amounts of fructose than K juices. Therefore, the differences found in the concentration of the individual sugars as in glucose, and especially in fructose, which has the highest sweetness perception among the sugars present in orange juice, might have an influence on the sweetness of the juice and their organoleptic quality.

**Figure 7:**
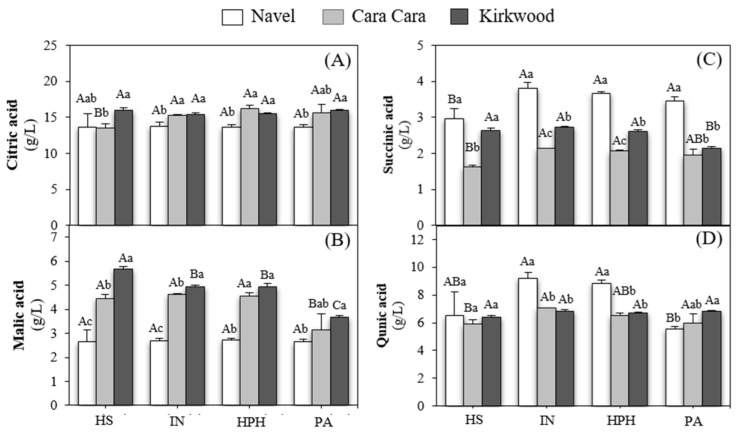
Content of citric acid (**A**), malic acid (**B**), succinic acid (**C**), and quinic acid (**D**) hand-squeezed (HS), industrial (IN), HPH, and pasteurized (PA) juices of NF, CC, and K orange. Uppercase letters indicate significant differences between different type of juices for the same variety, and lowercase letters indicate significant differences between varieties for the same type of juice one-way ANOVA (*p* < 0.05).

The main organic acids detected in all orange juices in order of abundance were: citric acid (13.52–16.02 g/L), quinic acid (5.65–9.17 g/L), malic acid (2.66–5.68 g/L), and succinic acid (1.61–3.81 g/L) (Figure 7).

Between these organic acids, citric acid is the largest contributor to the acidity taste in orange juice and accounts for 46.8–58.5% of total organic acids in all juices. The ratio of citric acid to total organic acids found in our study is similar to that reported by Albertini et al. [86] for other sweet oranges. In this work, consistent differences were observed in the concentration of individual acids between the juices of N and the two red-fleshed juices, which is a factor contributing to the characteristic acidity taste of orange juices and the organoleptic quality [87]. Particularly, the composition of organic acids in CC and K juices in comparison with N juices was characterized by a slightly higher amount of citric acid (10–20%) (Figure 7A), a notably higher content of malic acid (Figure 7B) and lower levels of succinic acid (Figure 7C). Minor variations in the content of organic acids between the HS and the industrial juice were detected. Thus, the type of juice squeezing did not affect the concentration of organic acids. The HPH and pasteurization treatments barely affected the concentration of organic acids in all the varieties, and only the pasteurization significantly reduced the content of malic acid in CC and K and around 40% that of quinic acid in N (Figure 7). These results agree with those described by other authors, which suggested that high pressures and thermal treatments did not substantially modify these compounds [29,85].

In general, the juices of both red-fleshed oranges presented a similar organic acids profile, but concentrations of malic and succinic were higher and lower, respectively than in the blond orange juices. Thus, these differences in total organic acids and their individual ratio could have an important effect on the sensory characteristics of the red-fleshed orange juices.

### 3.8. Multivariate Analysis of Chemical Composition and Antioxidant Capacity in Hand-Squeezed, Industrial, HPH, and Pasteurized Juices

**Figure 8:**
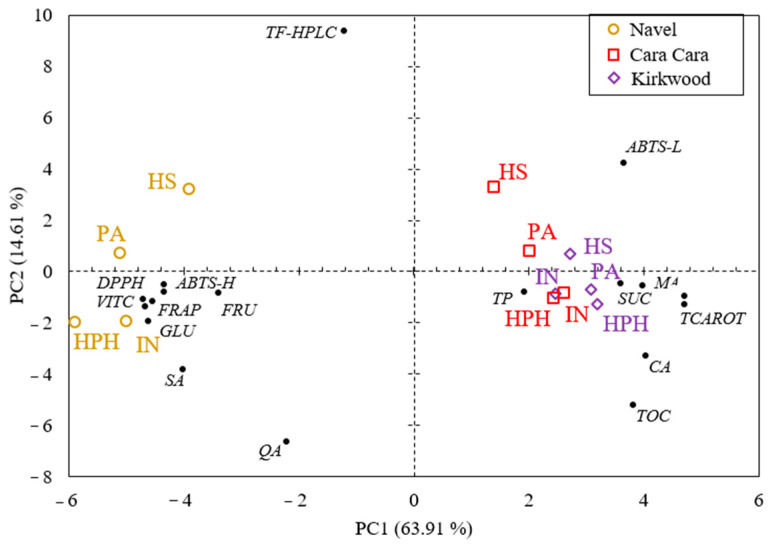
Principal component analysis (PCA) of total carotenoids (TCAROT), vitamin C (VITC), tocopherols (TOC), total phenolics (TP), total flavonoids by HPLC (TF-HPLC), sucrose (SUC), glucose (GLU), fructose (FRU), citric acid (CA), malic acid (MA), succinic acid (SA), quinic acid (QA) and antioxidant capacity (DPPH, FRAP, ABTS-H, and ABTS-L) in hand-squeezed (HS), industrial (IN), high-pressure homogenized (HPH) and pasteurized (PA) juices of N (yellow circles), CC (red squares) and K (purple diamonds) orange.

A principal component analysis (PCA) was performed to study the variability between the varieties and the different types of juices, considering the concentration of all the compounds analyzed and the antioxidant capacity (Figure 8). The first two components (PCs) explained 78.52% of the total variance of the data. The PC1 (63.91%) discriminates samples according to the concentration of compounds and antioxidant values. Thus, for each variety, the juices obtained by both extraction methods and treatments clustered together, indicating that minor differences were found between different types of juices. Furthermore, the juices of CC and K clustered very close, which means that the parameters considered in this study do not discriminate between the juices of both red-fleshed oranges, but they are clearly separate from those of N. In other terms, the juices of CC and K were characterized, in order from highest to lowest variance contribution, by higher levels of carotenoids, citric acid, malic acid, tocopherols, ABTS-L, sucrose, and total phenolic (Figure 8; Supplementary Table S1). Instead, the juices from N are distinguished by DPPH, FRAP, glucose, vitamin C, ABTS-H, succinic acid, and fructose (Figure 8; Supplementary Table S1). On the other hand, the PC2 (14.61%) separates samples principally by the content of total flavonoids by HPLC (TF-HPLC). This observation explained the fact that the HS juices, especially those of N and CC, grouped slightly separately regarding the other juices since their concentration of total flavonoids (TF-HPLC) was significantly higher. Considering the exhaustive characterization of the juices developed above and the results provided by the PCA, we might conclude that the concentration of carotenoids is the variable that contributes the most to the total variance and discriminates largely between the juices of N and the red-fleshed varieties.

### 3.9. Composition of Bioactive Compounds and Antioxidant Capacity of Navel Foios, Cara Cara, and Kirkwood Industrial Juice By-Product

**Figure 9:**
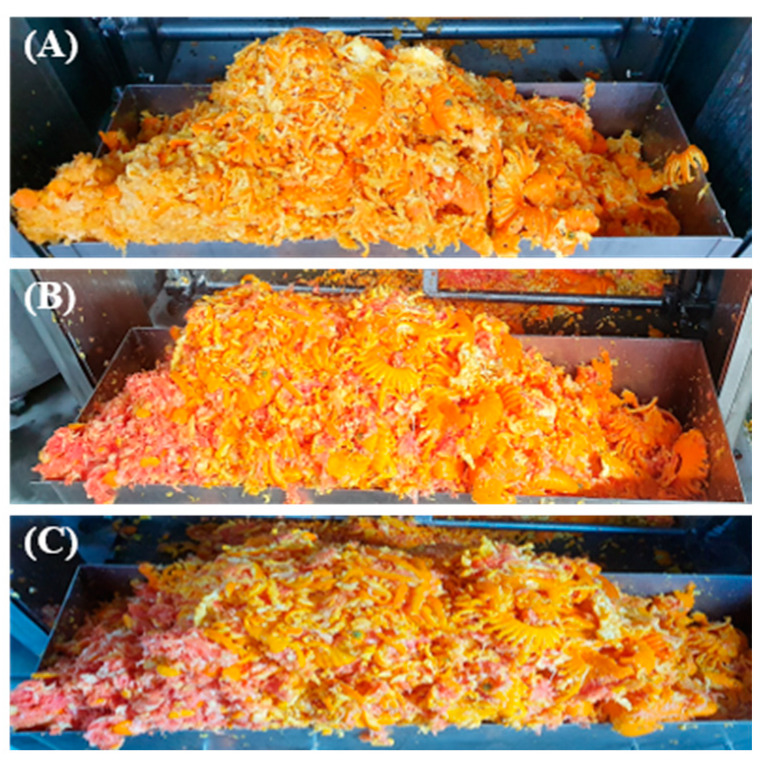
Image of the orange by-product of the standard variety Nave (**A**) and the red-fleshed varieties Cara Cara (**B**) and Kirkwood (**C**) generated during the industrial juice extraction.

In the present work, we carried out a comprehensive analysis of the composition of the main bioactive compounds and antioxidant capacity of waste generated during the industrial juice squeezing of the standard variety N and the red-fleshed orange CC and K (Figure 9). We examined the composition of carotenoids and the contents of vitamin C, tocopherols, total phenolics, and flavonoids, as well as the hydrophilic (DDPH, FRAP, and ABTS-H) and lipophilic (ABTS-L and SOAC) antioxidant capacity of the lyophilized waste generated from the three varieties. The total concentration of carotenoids in the by-product of N was 132.22 µg/g DW, while both red-fleshed varieties presented a concentration 10-fold higher, 1323.81 µg/g DW and 1344.56 µg/g DW in CC and K, respectively (Table 5).

In addition, the carotenoid composition of the by-products of the three varieties was markedly different. The carotenoid profile of each variety resembled that described for the corresponding juice (Section 3.2) and pulp [15,20,21,22,29,31]. The by-product from N is characterized by a large content of β,β-xanthophylls, mainly violaxanthin (47.15 µg/g DW) and luteoxanthin (18.67 µg/g DW), and the carotenes phytoene (29.72 µg/g DW) and phytofluene (16.39 µg/g DW). In contrast with the low proportion of the colorless phytoene and phytofluene in the pulp and juice of N oranges, these carotenes accounted for nearly 35% of total carotenoids in the by-product of N. Thus, it is likely that this proportion of colorless carotenes might come from the peel of the fruit (flavedo and albedo), which is a rich source of carotenoids [15,20,22].

The carotenoid content of the by-product of both red-fleshed oranges is mainly composed of large amounts of phytoene and phytofluene (87%), followed by lycopene (8–10%). β,β-Xanthophylls content was about half that found in N and accounted for about 3.5% of total carotenoids. It is worth mentioning that the concentration of phytoene and phytofluene in the by-product of both red-fleshed oranges was about 30- and 10 times, respectively, higher than in the by-product of the N orange (Table 5).

In addition, it is interesting to mention that β-carotene was also detected in the by-product of the red-fleshed oranges (0.6% of the total content), which determined a 50% increase in the value of retinol equivalents with respect to the N by-product (Table 5).

Important amounts of other bioactive compounds were also detected in the orange by-products of the three varieties (Table 6). The concentration of vitamin C found in N (151.87 mg/100 g DW), and CC (141.83 mg/100 g DW) was significantly higher than in K (99.90 mg/100 g DW). The levels of tocopherols in the three varieties ranged from 128.60–148.16 µg/g DW, with CC being the richest (Table 6). The isoforms α- and γ-tocopherol were identified in the by-product of all samples, being α-tocopherol the main in the three varieties, accounting for 86–88% of total tocopherols. The tocopherol composition observed in the by-product is similar to that described for the flavedo of mature orange fruits [47]. In the pulp of sweet oranges, γ-tocopherol is barely detectable, and concentrations of total tocopherols are very low (≤2%) [31,47]. Hence, it is reasonable to assume that the content of tocopherols in the orange by-product mainly comes from flavedo tissue. The concentration of total phenolic was significantly higher in N (2174.56 mg GAE/100 g DW) than in the red-fleshed CC (1901.64 mg GAE/100g DW) and K (1886.40 mg GAE/100 g DW). The content of flavonoids was similar between N (1548.96 mg HesE/100 g DW) and CC (1516.14 mg HesE/100 g DW) and slightly lower in K (1436.82 mg GAE/100 g DW). In general, it seems that the juice processing by-product of N contains larger amounts of water-soluble antioxidant compounds such as vitamin C, phenolic, and flavonoids than CC and K. This is consistent with the fact that the evaluation of the HAC determined by the FRAP and ABTS-H showed higher values of N orange by-product than those of CC and K (Table 6). Contrarily, ABTS-L showed higher capacity in the processing by-products of both red-fleshed varieties than in N (Table 6). Additionally, in order to quantify the quenching singlet oxygen capacity in these by-products, the SOAC analysis was performed. The K and CC by-products showed between 1.5 and 2 times higher SOAC values than N. Thus by-products of both red-fleshed varieties were more efficient singlet oxygen scavengers than N (Table 6). Carotenoids are recognized as efficient quenchers of singlet oxygen and other reactive oxygen species [88]. The relationship between the concentration of carotenoids and the capacity of quenching singlet oxygen (SOAC) in citrus fruits has been established [50], and our results corroborate those in which the pulp of the red-fleshed fruit had higher SOAC than the ordinary Navel orange [31].

Different studies have addressed the physico-chemical characterization of citrus waste and its importance for the revalorization in many applications, such as biofuel, biorefinery, compost, production, animal feed, essential oils, and extraction of pectin and other compounds with biological activity [42,43,89,90].

In this regard, the by-products from the red-fleshed oranges CC and K represent a higher added-value by-product compared to that of N due to the large concentration of carotenoids, especially phytoene, phytofluene, lycopene (which is not present in standard blond oranges), tocopherols and their enhanced antioxidant capacity against singlet oxygen. Altogether, the red-fleshed orange processing by-product is a promising source of bioactive compounds with high potential in the food and nutraceutical industry.

## 4. Conclusions

In this study, we investigated the composition of nutrients and bioactive compounds, and antioxidant capacity in juices of two red-fleshed oranges, CC and K, in comparison with the standard blond Navel orange, obtained by two extraction methods and two different treatments. Since the fruit of the common Navel and the red-fleshed varieties were harvested in different locations, we cannot discard the effect of growing conditions on the composition of the fresh juices. For the three orange varieties, the hand and industrial juice squeezing showed similar efficiency in extracting the compounds from orange fruits, with some exceptions. The treatments HPH (150 MPa/55 °C/min) and pasteurization (85 °C/30 s) rendered red-fleshed orange juices with a similar composition to the corresponding fresh juices. The most remarkable difference in CC and K compared with N juice was the 3- to 6-times higher carotenoid content, phytoene, and phytofluene, the main carotenoids. The HPH and pasteurization affected the carotenoid concentration of each variety variably, mainly due to the different compositions of the juices. Vitamin C was substantially higher in Navel, while both red-fleshed varieties presented higher amounts of tocopherols. Additionally, vitamin C was the main contributor to the hydrophilic antioxidant capacity, whereas the higher lipophilic antioxidant capacity of CC and K could be associated with the large concentrations of carotenes. Finally, the orange by-product of the red-fleshed oranges generated during industrial juice extraction is greatly enriched in carotenoids, and its lipophilic fraction has a high antioxidant capacity against singlet oxygen.

The increasing demand of consumers for healthy and environmentally friendly products leads the food industry to search for sustainable processes and foods with high added value. In this context, juices from red-fleshed orange varieties, fresh hand-squeezed or industrial processed, represent a rich source of dietary carotenoids and enhanced antioxidant capacity without significant detrimental effects on other bioactive compounds. Therefore, the juices from red pulp oranges could provide increased benefits for human nutrition and health in comparison with standard orange juice. In addition, the orange by-products from red-fleshed oranges show higher potential for revalorization as functional ingredients for designing healthy foods or other nutraceutical industrial applications.

## Figures and Tables

**Table 1:** Soluble solids (SS), titratable acidity (TA) maturity index (MI) of Navel, Cara Cara, and Kirkwood oranges.

Parameters	Navel	Cara Cara	Kirkwood
SS (°Brix)	11.45 ± 0.07 a	9.97 ± 0.06 b	11.17 ± 0.06 a
TA (mg/CA 100 mL)	0.82 ± 0.05 a	0.70 ± 0.06 a	0.91 ± 0.06 a
MI (SS/TA)	14.08 ± 0.94 a	14.36 ± 1.04 a	12.35 ± 0.87 a

Lowercase letters indicate significant differences between varieties by one-way ANOVA (*p* < 0.05).

**Table 2:** Color coordinate *L* and the ratio *a/b* of HS (hand-squeezed), industrial, HPH (high-pressure homogenized), and pasteurized juices of Navel, Cara Cara, and Kirkwood oranges.

	Navel		Cara Cara		Kirkwood	
Juice	*L*	*a/b*	*L*	*a/b*	*L*	*a/b*
HS	45.01 ± 0.05 Ba	0.15 ± 0.03 Ac	40.48 ± 0.01 Bb	0.32 ± 0.02 Ab	40.97 ± 0.33 Bb	0.37 ± 0.01 Aa
Industrial	55.50 ± 0.04 Aa	0.03 ± 0.01 Bb	47.12 ± 0.07 Ac	0.24 ± 0.01 Ba	51.21 ± 0.38 Ab	0.21 ± 0.01 Ba
HPH	56.10 ± 0.06 Aa	−0.01 ± 0.01 Bb	49.78 ± 0.30 Ab	0.19 ± 0.04 Ba	49.40 ± 0.09 Ab	0.24 ± 0.02 Ba
Pasteurized	56.25 ± 0.04 Aa	0.02 ± 0.01 Bb	49.19 ± 0.04 Ab	0.22 ± 0.01 Ba	49.40 ± 0.09 Ab	0.20 ± 0.01 Ba

Uppercase letters indicate significant differences between different types of juices for the same variety, and lowercase letters indicate significant differences between varieties for the same type of juice by one-way ANOVA (*p* < 0.05).

**Table 3:** Carotenoid content and composition (µg/mL) of HS (hand-squeezed), industrial, HPH (high-pressure homogenized), and pasteurized juices of Navel, Cara Cara, and Kirkwood oranges.

	Navel				Cara Cara				Kirkwood			
Carotenoids	HS	Industrial	HPH	Pasteurized	HS	Industrial	HPH	Pasteurized	HS	Industrial	HPH	Pasteurized
Phytoene	0.14 ± 0.03 Bb	0.38 ± 0.02 Ac	0.24 ± 0.01 ABb	0.15 ± 0.05 Bc	14.99 ± 2.37 Ba	23.12 ± 0.68 Aa	20.44 ± 1.15 Aa	20.52 ± 0.20 Aa	18.00 ± 0.85 Ba	18.35 ± 0.22 Bb	22.14 ± 0.60 Aa	19.97 ± 0.04 Bb
Phytfl.	N.D.	0.09 ± 0.01 Ac	0.07 ± 0.02 Ab	0.06 ± 0.01 Ac	3.48 ± 0.61 Ca	5.02 ± 0.19 ABa	4.58 ± 0.24 Ba	5.59 ± 0.01 Aa	4.23 ± 0.10 BCa	3.99 ± 0.01 Cb	4.89 ± 0.18 Aa	4.50 ± 0.01 ABb
ζ-carotene	0.07 ± 0.02 Ab	0.08 ± 0.01 Ac	0.05 ± 0.01 Ab	0.07 ± 0.01 Ac	0.24 ± 0.05 Ba	0.26 ± 0.03 Ba	0.23 ± 0.02 Ba	0.34 ± 0.01 Aa	0.27 ± 0.01 Aa	0.17 ± 0.01 Cb	0.22 ± 0.01 Ba	0.28 ± 0.01 Ab
Neur.	N.D.	N.D.	N.D.	N.D.	0.06 ± 0.02 Ba	0.22 ± 0.01 Aa	0.08 ± 0.06 Ba	0.09 ± 0.01 Ba	0.07 ± 0.03 Aa	0.18 ± 0.01 Ab	0.11 ± 0.05 Aa	0.08 ± 0.03 Aa
Lycopene	N.D.	N.D.	N.D.	N.D.	1.30 ± 0.27 Ba	2.29 ± 0.18 Aa	1.95 ± 0.03 ABb	2.30 ± 0.03 Aa	1.49 ± 0.52 Ba	1.56 ± 0.06 Bb	2.82 ± 0.24 Aa	2.39 ± 0.19 ABa
Lutein	0.29 ± 0.06 Aa	0.35 ± 0.02b	0.30 ± 0.03 Aa	0.30 ± 0.06 Aa	0.12 ± 0.02 Cb	0.18 ± 0.01 Bc	0.17 ± 0.01 Bb	0.24 ± 0.01 Aa	0.22 ± 0.01 Ba	0.54 ± 0.02 Aa	0.23 ± 0.03 Bab	0.21 ± 0.01 Ba
β-carotene	N.D.	N.D.	N.D.	N.D.	0.23 ± 0.05 Ca	0.31 ± 0.01 BCa	0.38 ± 0.03 ABb	0.47 ± 0.03 Aa	0.29 ± 0.02 Ca	0.34 ± 0.02 BCa	0.45 ± 0.02 Aa	0.37 ± 0.01 Bb
β-Crypto.	0.65 ± 0.14 Aa	0.91 ± 0.05 Aa	0.69 ± 0.05 Ab	0.66 ± 0.01 Ac	0.55 ± 0.11 BCa	0.52 ± 0.01 Cc	0.73 ± 0.02 Bab	0.96 ± 0.01 Aa	0.64 ± 0.04 Ca	0.67 ± 0.02 BCb	0.85 ± 0.04 Aa	0.79 ± 0.04 ABb
Zeax.	0.14 ± 0.04 Aa	0.18 ± 0.01 Aa	0.16 ± 0.01 Ab	0.15 ± 0.04 A	0.12 ± 0.04 Cb	0.15 ± 0.01 Ca	0.21 ± 0.01 Ba	0.29 ± 0.02 Aa	0.26 ± 0.09 Aa	0.26 ± 0.05 Aa	0.20 ± 0.02 Aa	0.30 ± 0.05 Aa
Anthx.	1.00 ± 0.13 Aa	0.75 ± 0.02 ABa	0.63 ± 0.04 Bb	0.52 ± 0.03 Bc	0.47 ± 0.08 Bb	0.50 ± 0.08 Bb	0.59 ± 0.05 Bb	1.10 ± 0.01 Aa	1.25 ± 0.03 Aa	0.66 ± 0.01 Cb	0.95 ± 0.01 Ba	0.69 ± 0.02 Cb
Viol.	4.25 ± 0.31 Aa	4.25 ± 0.45 Aa	2.84 ± 0.06 Ba	2.70 ± 0.08 Ba	2.80 ± 0.48 ABb	3.36 ± 0.10 Aa	2.49 ± 0.15 Ba	2.30 ± 0.32 Bab	2.41 ± 0.04 Ab	2.32 ± 0.08 Ab	2.04 ± 0.03 Bb	1.93 ± 0.02 Bb
Luteox.	0.62 ± 0.06 Ca	1.44 ± 0.09 Aa	0.80 ± 0.20 BCa	1.14 ± 0.01 Ba	0.29 ± 0.10 Cb	0.65 ± 0.01 B	0.62 ± 0.01 B	0.87 ± 0.03 Aab	0.20 ± 0.06 Cb	0.77 ± 0.05 A	0.48 ± 0.01 Bb	0.69 ± 0.07 Ab
Mutato.	0.11 ± 0.01 Ca	0.52 ± 0.07 Aa	0.28 ± 0.01 Ba	0.45 ± 0.01 Aa	0.10 ± 0.01 Ca	0.25 ± 0.01 Bb	0.25 ± 0.02 Ba	0.41 ± 0.03 Aa	0.06 ± 0.01 Cb	0.45 ± 0.01 Aa	0.33 ± 0.05 Ba	0.44 ± 0.01 Aa
Total carot.	7.28 ± 0.54 Bb	8.94 ± 0.24 Ac	6.05 ± 0.42 Bb	6.21 ± 0.12 Bc	24.76 ± 4.20 Ba	36.88 ± 0.82 Aa	32.73 ± 1.84 Aa	35.47 ± 0.12 Aa	29.38 ± 0.48 Ca	30.27 ± 0.25 Cb	35.67 ± 0.17 Aa	32.64 ± 0.28 Bb
RE	0.05 ± 0.01 Ab	0.08 ± 0.01 Ab	0.06 ± 0.0.4 Ab	0.06 ± 0.01 A	0.09 ± 0.02 Ba	0.10 ± 0.01 Ba	0.12 ± 0.01 ABa	0.16 ± 0.02 Aa	0.10 ± 0.03 Aa	0.11 ± 0.01 Aa	0.15 ± 0.02 Aa	0.13 ± 0.02 Aa

Phytfl., Phytofluene; Neur., Neurosporene; Zeax., Zeaxanthin; Anthx., Antheraxanthin; Viol., Violaxanthin; Luteox., Luteoxanthin; Mutato., Mutatoxanthin. Uppercase letters indicate significant differences between different types of juices for the same variety, and lowercase letters indicate significant differences between varieties for the same type of juice by one-way ANOVA (*p* < 0.05). β-crypto: β-cryptoxanthin. N.D: not detected. RE: retinol equivalents.

**Table 4:** Flavonoid content and composition (mg/100 mL) of HS (hand-squeezed), industrial, HPH (high-pressure homogenized), and pasteurized juices of Navel, Cara Cara, and Kirkwood oranges.

	Navel				Cara Cara				Kirkwood			
Flavonoids	HS	Industrial	HPH	Pasteurized	HS	Industrial	HPH	Pasteurized	HS	Industrial	HPH	Pasteurized
Rutin	1.20 ± 0.06 Ba	1.46 ± 0.01 Aa	1.53 ± 0.06 Aa	1.57 ± 0.13 Aa	1.09 ± 0.03 Ba	1.12 ± 0.02 Bb	1.35 ± 0.02 Ab	1.22 ± 0.08 ABab	1.11 ± 0.04 Aa	1.17 ± 0.13 Aab	1.08 ± 0.01 Ac	1.12 ± 0.01 Ab
Eriocitrin	0.36 ± 0.01 Aa	0.40 ± 0.01 Aa	0.41 ± 0.01 Ab	0.43 ± 0.04 Aa	0.40 ± 0.05 Ba	0.50 ± 0.01 ABa	0.57 ± 0.02 Aa	0.41 ± 0.03 Ba	0.48 ± 0.03 Aa	0.49 ± 0.06 Aa	0.53 ± 0.01 Aa	0.39 ± 0.01 Ba
Narirutin	5.72 ± 0.19 Aa	5.42 ± 0.01 Aa	5.57 ± 0.18 Aa	5.81 ± 0.55 Aa	4.14 ± 0.08 Bb	4.05 ± 0.03 Bb	4.81 ± 0.07 Ab	4.49 ± 0.33 ABb	4.01 ± 0.20 Bb	4.52 ± 0.54 ABab	4.15 ± 0.03 ABc	4.27 ± 0.01 Ab
Naringin	0.08 ± 0.01 Aa	0.10 ± 0.01 Aa	0.09 ± 0.01 Aa	0.09 ± 0.01 Aa	0.09 ± 0.01 Aa	0.08 ± 0.01 Aa	0.10 ± 0.01 Aa	0.10 ± 0.01 Aa	0.09 ± 0.01 Aa	0.11 ± 0.01 Aa	0.10 ± 0.01 Aa	0.10 ± 0.01 Aa
Hesperidin	17.57 ± 1.04 Aa	10.88 ± 0.23 Ba	10.67 ± 0.54 Ba	12.29 ± 1.15 Ba	17.32 ± 0.07 Aa	11.92 ± 0.01 Ba	11.54 ± 0.28 Ba	13.68 ± 1.14 Ba	15.53 ± 2.04 Aa	11.51 ± 2.91 Aa	10.80 ± 0.07 Aa	11.30 ± 0.84 Aa
Dydimin	1.12 ± 0.02 Aa	0.63 ± 0.04 Ba	0.64 ± 0.04 Bb	0.69 ± 0.06 Bb	1.21 ± 0.06 Aa	0.74 ± 0.03 Ca	0.86 ± 0.06 Ca	1.04 ± 0.01 Ba	0.80 ± 0.06 Ab	0.66 ± 0.07 Aa	0.73 ± 0.03 Aab	0.69 ± 0.03 Ab
Total	26.05 ± 1.33 Aa	18.89 ± 0.29 Ba	18.91 ± 0.84 Ba	20.89 ± 1.94 Ba	24.25 ± 0.29 Aa	18.41 ± 0.09 Ba	19.23 ± 0.45 Ba	20.94 ± 1.70 ABa	22.03 ± 2.37 Aa	18.46 ± 3.73 Aa	17.39 ± 0.15 Ab	17.87 ± 0.88 Aa

Uppercase letters indicate significant differences between different type of juices for the same variety, and lowercase letters indicate significant differences between varieties for the same type of juice by one-way ANOVA (*p* < 0.05).

**Table 5:** Carotenoid content and composition (µg/g DW) of Navel, Cara Cara, and Kirkwood orange by-products.

Parameters	Navel	Cara Cara	Kirkwood
Phytoene	29.72 ± 10.91 b	998.09 ± 131.41 a	988.94 ± 31.55 a
Phytoflluene	16.39 ± 2.09 b	164.01 ± 35.36 a	166.72 ± 5.65 a
ζ-carotene	4.91 ± 0.67 a	3.57 ± 1.56 ab	2.23 ± 0.14 b
Neurosporene	N.D.	3.57 ± 0.07 b	7.82 ± 0.27 a
Lycopene	N.D.	104.36 ± 4.16 b	123.25 ± 9.92 a
Lutein	0.93 ± 0.05 a	N.D.	0.57 ± 0.08 b
β-carotene	N.D.	7.84 ± 2.30 a	7.74 ± 0.85 a
β-cryptoxanthin	5.73 ± 0.50 a	4.09 ± 0.58 a	3.27 ± 0.06 b
Zeaxanthin	1.08 ± 0.13 a	N.D.	1.39 ± 0.15 a
Antheraxanthin	4.37 ± 6.18	N.D.	N.D.
Violaxanthin	47.15 ± 1.58 a	30.70 ± 1.24 b	33.43 ± 0.27 b
Luteoxanthin	18.67 ± 0.64 a	7.12 ± 0.38 b	7.09 ± 0.34 b
Mutatoxanthin	3.30 ± 0.14 a	0.46 ± 0.21 c	2.09 ± 0.13 b
Total carotenoids	132.22 ± 8.73 b	1323.81 ± 184.42 a	1344.56 ± 47.29 a
RE	0.96 ± 0.12 b	1.66 ± 0.19 a	1.56 ± 0.17 a

Lowercase letters indicate significant differences between orange by-products of each variety by one-way ANOVA (*p* < 0.05). N.D: not detected. R.E: retinol equivalents.

**Table 6:** Content of vitamin C, tocopherols, total phenolics, total flavonoids, and antioxidant capacity (DPPH, FRAP, ABTS-H, ABTS-L, and SOAC) of Navel, Cara Cara, and Kirkwood orange by-products.

Parameters	Navel	Cara Cara	Kirkwood
Vitamin C (mg/100 g DW)	151.87 ± 4.62 a	141.83 ± 5.07 a	99.90 ± 2.80 b
Tocopherols (µg/g DW)	128.60 ± 4.69 b	148.16 ± 0.92 a	139.12 ± 5.97 ab
Total phenolics (mg GAE/100 g DW)	2174.56 ± 4.85 a	1901.64 ± 75.51 b	1886.40 ± 78.62 b
Total flavonoids (mg HesE/100 g DW)	1548.96 ± 42.98 a	1516.14 ± 16.18 a	1436.82 ± 11.92 b
DPPH (mg AsAE/100 g DW)	179.16 ± 5.90 a	167.72 ± 6.15 ab	154.09 ± 3.01 b
FRAP (mg AsAE/100 g DW)	224.04 ± 12.23 a	203.91 ± 0.97 b	188.01 ± 4.38 c
ABTS-H (TEAC/100 g DW)	1237.61 ± 52.42 a	1048.44 ± 54.72 b	1049.28 ± 42.93 b
ABTS-L (TEAC/100 g DW)	155.37 ± 1.30 b	172.92 ± 1.60 a	171.52 ± 2.10 a
SOAC ^a^	0.47 ± 0.02 c	0.79 ± 0.04 b	0.92 ± 0.04 a

GAE: gallic acid equivalents. HesE: hesperidin equivalents. AsAE: ascorbic acid equivalents. TEAC: trolox equivalent antioxidant capacity. SOAC: singlet oxygen absorption capacity. Lowercase letters indicate significant differences between orange by-products of each variety by one-way ANOVA (*p* < 0.05). ^a^ Relative SOAC values based on g/L unit.

## Data Availability

All the data are contained in the article and Supplementary Materials.

## Supplementary Materials

The following supporting information can be downloaded at: https://www.mdpi.com/article/10.3390/foods12020400/s1, Table S1: Percentage of the contribution of variables to the total variance of principal components 1 and 2.
